# Form‐Specific and Probabilistic Environmental Risk Assessment of 3 Engineered Nanomaterials (Nano‐Ag, Nano‐TiO_2_, and Nano‐ZnO) in European Freshwaters

**DOI:** 10.1002/etc.5146

**Published:** 2021-08-04

**Authors:** Hyunjoo Hong, Véronique Adam, Bernd Nowack

**Affiliations:** ^1^ Empa, Swiss Federal Laboratories for Materials Science and Technologies, Technology and Society Laboratory St. Gallen Switzerland

**Keywords:** Risk assessment, Nanomaterials, Nanoecotoxicology

## Abstract

The release of engineered nanomaterials (ENMs) to the environment necessitates an assessment of their environmental risks. The currently available environmental risk assessments (ERA) for ENMs are based on an analysis of the total flows of a specific ENM to the environment and on ecotoxicity studies performed with pristine ENMs. It is known that ENMs undergo transformation during product use and release and in technical systems such as wastewater treatment. The aim of the present study was therefore to perform an ERA of 3 ENMs (nano‐Ag, nano‐TiO_2_, and nano‐ZnO) based on a form‐specific release model and a form‐specific analysis of ecotoxicological data. Predicted environmental concentration values were derived using a form‐specific material flow model. Species sensitivity distributions were used to derive predicted‐no‐effect concentrations (PNECs) for the pristine ENMs and for dissolved and transformed Ag and ZnO. For all ENMs, the matrix‐embedded form was included in the assessment. A probabilistic assessment was applied, yielding final probability distributions for the risk characterization ratio (RCR). For nano‐Ag, the form‐specific assessment resulted in a decrease of the mean RCR from 0.061 for the approach neglecting the different release forms to 0.034 because of the much lower PNEC of transformed Ag. Likewise, for nano‐ZnO, the form‐specific approach reduced the mean RCR from 1.2 to 0.86. For nano‐TiO_2_, the form‐specific assessment did not change the mean RCR of 0.026. This analysis shows that a form‐specific approach can have an influence on the assessment of the environmental risks of ENMs and that, given the availability of form‐specific release models, an updated ERA for ENMs can be performed. *Environ Toxicol Chem* 2021;40:2629–2639. © 2021 The Authors. *Environmental Toxicology and Chemistry* published by Wiley Periodicals LLC on behalf of SETAC.

## INTRODUCTION

Engineered nanomaterials (ENMs) are widely used in many commercial products and applications and can be released into the environment during their whole life cycle. Material flow analysis (MFA) is a method that can quantify the environmental emissions of substances throughout the life cycle of products (Brunner and Rechberger 2004). A suite of MFA models has been developed over the last 12 yr to predict the flows of ENMs to the environment (Hendren et al. 2013; Baalousha et al. 2016; Wigger et al. 2020a). These models consider as input data the production of ENMs, the use of ENMs in various product categories, and transfer coefficients describing the proportion of ENMs flowing from the product categories to technical compartments such as the sewer system, wastewater‐treatment plants, recycling, incineration, landfills, and environmental compartments such as surface water and soils.

All models until 2018 only considered a “generic ENM,” tracking the complete mass of the produced ENM from production to the final sinks. No distinction was made between different nanoforms of the same material and potential changes that occur during use and release. Mitrano et al. (2015) reviewed the key use‐related aging and transformation processes affecting ENMs. Processes resulting in transformation of the released materials include photochemical transformations, oxidation and reduction, dissolution, precipitation, adsorption and desorption, combustion, biotransformation, and abrasion. Depending on product use and the susceptibility of a specific ENM to these transformation reactions, the released ENM can or cannot be similar to the original, pristine ENM. An example is nano‐Ag contained in textiles, where many studies have shown that dissolution and transformation occur even before use, during use in the presence of sweat, during washing, and within wastewater‐treatment plants. A suite of transformation products such as dissolved Ag, AgCl, and Ag_2_S has been reported to be released (Mitrano et al. 2015). Just tracking the flows of ENMs without considering these reactions will lead to a wrong prediction of the type of material actually present in a technical or environmental compartment. Some models have included transformation during wastewater treatment or incineration as a process leading to a flow of materials out of the system's boundary (see Gottschalk et al. 2009; Sun et al. 2014). Adam et al. (2018) were the first to systematically assess the flows of different released forms throughout the complete system. The ENM flows were separated into pristine, dissolved, transformed, matrix‐embedded, and product‐embedded. Models were first published for nano‐Ag and nano‐TiO_2_ (Adam et al. 2018) and further extended to nano‐ZnO (Adam et al. 2021). Nano‐Ag was modeled to be released to surface water and soil mainly as transformed nano‐Ag (46 and 79%, respectively), whereas the release to air was found to be mostly as pristine and matrix‐embedded nano‐Ag (42 and 40%, respectively). Nano‐TiO_2_, on the other hand, was predominantly released to air, soil, and water in pristine form (80, 91, and 97%; Adam et al. 2018). These results show that for some ENMs the transformation reactions cannot be neglected and that mass flows of different forms need to be considered. Wigger and Nowack (2019) extended this approach and included the initial nanoform in the flow assessment. The authors separated the flows of different nanoforms of the same ENM, for example, anatase and rutile nano‐TiO_2_, single‐ and multiwalled carbon nanotubes (CNTs), or α‐ and γ‐Al_2_O_3_.

Knowledge about the various forms of an ENM is important because the different forms can have very different effects on organisms. Sulfidation of nano‐Ag, for example, results in a significant decrease in toxicity (Levard et al. 2012). Looking at the available studies on effects of transformation on toxicity, Lehutso et al. (2020) and Zhang et al. (2018) showed both increased and decreased toxicity for different transformation reactions. However, the available environmental risk assessment (ERA) studies for ENMs have so far performed the hazard assessment solely using data obtained for the pristine form, as detailed in a recent review by Wigger et al. (2020a).

Of the 3 transformed forms considered by Adam et al. (2018), there is clearly a lot of hazard information available for the dissolved metal ions because knowledge about dissolved metals forms the basis for metal risk assessment (Mebane et al. 2020). As reviewed by Zhang et al. (2018), there are also some data available for transformed ENMs. Many ENMs are applied in products in the form of nanocomposites, and several reviews have highlighted that release from these nanocomposites is mainly in the form of matrix fragments containing the ENM (Froggett et al. 2014; Wohlleben and Neubauer 2016).

Amorim et al. (2018) showed for environmental organisms that the properties of the matrix drive the toxicity of the matrix‐embedded ENM. Also, Auffan et al. (2018) showed that CuO embedded in a matrix did not have any toxic effects at the tested concentration in a mesocosm experiment and that dissolved Cu was the major component determining the toxicity. A few toxicological studies conducted with matrix‐embedded ENMs have been carried out in vivo (Saber et al. 2012, 2018; Wohlleben et al. 2013; Ging et al. 2014; Smulders et al. 2014) and in vitro (Wohlleben et al. 2011; Kaiser et al. 2013; Mikkelsen et al. 2013). These studies showed that in most cases the toxicity of the matrix fragments was much less than that of the pristine particles; however, they all had the limitation that the comparison between the toxicity of the matrix particles containing ENMs and the ENM alone at the same concentration was often missing. Kaiser et al. (2013) and Smulders et al. (2014) argued that the toxic effects of the ENM containing matrix particles are dependent on the toxicity of the matrix. Schlagenhauf et al. (2015) not only were the first to quantify the amount of ENM released in matrix‐embedded form but also developed a method to quantify the concentration of ENM protruding from the surface of the fragments.

The aim of the present study was to characterize the environmental risk of 3 ENMs (nano‐Ag, nano‐TiO_2_, and nano‐ZnO) to the freshwater compartment in Europe considering the different forms of the released ENMs. The predicted environmental concentration (PEC) values of the different forms (pristine, dissolved, transformed, and matrix‐embedded) were taken from Adam et al. (2018, 2021). For the hazard assessment, we built an ecotoxicological data set for each of the different forms of the 3 ENMs and calculated probabilistic species sensitivity distributions (PSSDs) using the PSSD + method (Wigger et al. 2020b). Finally, the environmental risks were determined by calculating the risk characterization ratio (RCR) for each released form.

## METHODS

### Exposure assessment

The PEC values for pristine, dissolved, transformed, and matrix‐embedded ENMs were taken from the form‐specific MFA models of Adam et al. (2018) for nano‐TiO_2_ and nano‐Ag and from Adam et al. (2021) for nano‐ZnO. The present study adopts the definitions used in Adam et al. (2018). Nanoparticles (NPs) that are released without any transformation are defined as “pristine nanomaterials.” After dissolution of ENMs and the formation of dissolved ion, they are defined to be “any dissolved species released from an ENM.” When an ENM underwent chemical reactions, for example, sulfidation, the form of ENM is defined to be “transformed.” Lastly, the released ENMs embedded in a solid matrix are defined as a “matrix‐embedded” form, whereas product‐embedded ENMs are still contained in the product and have not been released in any form. There are 2 types of matrix‐embedded ENMs: ENMs that protrude on the surface of the ENM‐containing matrix particle (i.e., surface‐protruding ENM) and ENMs that are completely embedded in the matrix. The completely embedded ENMs are expected to be nontoxic because they are not bioavailable as long as the matrix does not degrade (Amorim et al. 2018). Because the present study aims to determine the risk induced by the ENMs and not by the matrix, only the protruding ENM are targeted in the exposure assessment. Adam et al. (2018, 2021) only provide data for the complete mass of the matrix‐embedded ENM. For that reason, the mass flows for matrix‐embedded ENMs provided by Adam et al. (2018, 2021) were multiplied by a conversion factor to obtain the mass of surface‐protruding ENM. This conversion factor was determined on the basis of an empirical study by Schlagenhauf et al. (2015), who quantified that the fraction of surface‐protruding CNT on the surface of released particles after abrasion from a polymer nanocomposite is 0.004. Because these are the only available empirical data to quantify this fraction, we used them, although they are valid for a different ENM and only for one specific polymer.

The PEC distribution of each form is derived from the probability distribution of the total input flows of the MFA by dividing the amounts of substances released into the freshwater compartment by the volume of this compartment in Europe. The volume was approximated based on the surface area and an average depth of freshwater within Europe (see Supplemental Data, Table S1).

### Hazard assessment

Ecotoxicological hazard data for pristine ENM in freshwaters were taken from Coll et al. (2016) for nano‐Ag and nano‐ZnO and from Wigger and Nowack (2019) for nano‐TiO_2_ and updated with newer data. A search was performed on Google Scholar to identify additional peer‐reviewed articles published before September 2019. For dissolved Ag and Zn, Garner et al. (2015) was taken as a basis for the literature search. For the transformed forms of nano‐Ag, ecotoxicity studies for AgS were used. For nano‐TiO_2_, ecotoxicity data were not required for dissolved and transformed TiO_2_ because TiO_2_ exists only in pristine and matrix‐embedded forms. The hazard of matrix‐embedded ENM was assumed to be the same as that of pristine ENM but considering the actual concentration of surface‐protruding ENM.

The selection criteria for determining the input data for the present study were based on Coll et al. (2016). However, additional criteria were considered to improve the quality of the hazard assessment: toxicity studies with pathogenic microorganisms (e.g., *Escherichia coli*) were excluded; when effects were measured at different time points, only the value from the last time point was considered; if both the lowest‐observed‐effect concentration (LOEC) and the no‐observed‐effect concentration (NOEC) values were available, the NOEC was chosen; and if several endpoints were reported, lethal effects were prioritized over other effects (e.g., growth, reproduction).

The obtained data points of the effect concentration were available in various types of dose descriptors, for instance, *x*% effect concentration (EC*x*), *x*% lethal concentration, LOEC, highest observed no‐effect concentration, and NOEC. Acute NOEC values were converted to chronic NOEC values by dividing the values by the relevant uncertainty factors (UFs): UF_t_ and UF_dd_. According to Wigger et al. (2020b), UF_t_ considers the exposure time of the experiment and thus converts acute toxicity into chronic toxicity; UF_dd_ transforms a specific dose descriptor to a NOEC value. The UF_t_ values provided by Wigger et al. (2020b) were complemented by including the types of species not considered in the previous study: amphibian, bacteria, hydroid, and ciliated protozoan (see Supplemental Data, section 2).

The predicted‐no‐effect concentration (PNEC) was calculated based on the transformed ecotoxicity values (i.e., chronic NOECs) using the PSSD + method (Wigger et al. 2020b). The first step of the PSSD + method is to compute the species‐specific NOEC probability distributions. The minimum and maximum values of each distribution are determined by the coefficient of variation for interlaboratory variations (30%) around the toxic values and the coefficient of variation of the UFs (50%). A Monte Carlo simulation with 10 000 iterations was performed within the ranges of species‐specific NOEC distributions to determine 10 000 NOEC values per species. The 10 000 PSSDs obtained were used to quantify the PNEC_*PSSD*+_ by extracting the hazardous concentration for 5% of species (HC5; European Chemicals Agency 2008). The HC5 was taken as the PNEC value.

If there were too few data points and species to construct a PSSD (fewer than 10 data points covering fewer than 8 taxonomic groups; European Chemicals Agency 2008), the PNEC_*deter*_ was deterministically calculated following the assessment factor method of the Registration, Evaluation, Authorisation, and Restriction of Chemicals guidance (European Chemicals Agency 2008).

### Environmental risk assessment

The risk of ENM was characterized by the RCR distribution. The RCR distribution was derived by dividing the PEC distribution by the PNEC_*PSSD+*_ distribution for each released ENM form. The PEC distribution was divided by a single value of PNEC_*deter*_ when the PNEC_*PSSD+*_ was not available. It should be noted that the risks of matrix‐embedded ENM were characterized by using the PEC distributions of surface‐protruding ENM and the PNEC of the pristine form.

The RCR of ENMs in the standard approach (generic ENM and PNEC of the pristine form) was approximated using Equation 1, based on the mean PEC and PNEC values from the present study:

(1) $${\text{RCR}}_{\text{standard}}\text{ = }\frac{{\text{PEC}}_{\text{pris}}{\text{ + PEC}}_{\text{diss}}{\text{ + PEC}}_{\text{trans}}{\text{ + PEC}}_{\text{surface}\text{‐}\text{protruding}}}{\text{PNEC of pristine ENM}}$$

In Equation 1, RCR_standard_, PEC_pris_, PEC_diss_, PEC_trans_, and PEC_surface‐protruding_ are the RCR derived from the standard approach and the PEC of pristine, dissolved, transformed, and surface‐protruding ENM, respectively.

The risk of ENMs considering all released forms is referred to as RCR_form‐specific_ and was calculated based on the assumption that the different forms of ENMs do not have any synergistic or antagonistic effects (European Chemicals Agency 2017).

(2) $${\text{RCR}}_{\text{form}-\text{specific}}{\text{ = RCR}}_{\text{pris}}{\text{ + RCR}}_{\text{diss}}{\text{ + RCR}}_{\text{trans}}{\text{ + RCR}}_{\text{surface}\text{‐}\text{protruding}}$$

In Equation 2, RCR_pris_, RCR_diss_, RCR_trans_, and RCR_surface‐protruding_ are the RCRs of pristine ENM, pristine nano‐ENM, transformed ENM, and surface‐protruding ENM, respectively.

## RESULTS

### Exposure assessment

The PECs of the different forms of nano‐Ag, nano‐TiO_2_, and nano‐ZnO are summarized in Supplemental Data, Table S4. The mean of the total concentration of the ENMs, which includes all released forms in the freshwater compartment (pristine, dissolved, transformed, and surface‐protruding ENM) are in the following order: nano‐Ag (0.0012 µg/L) < nano‐ZnO (0.17 µg/L) < nano‐TiO_2_ (0.70 µg/L).

**Figure 1:**
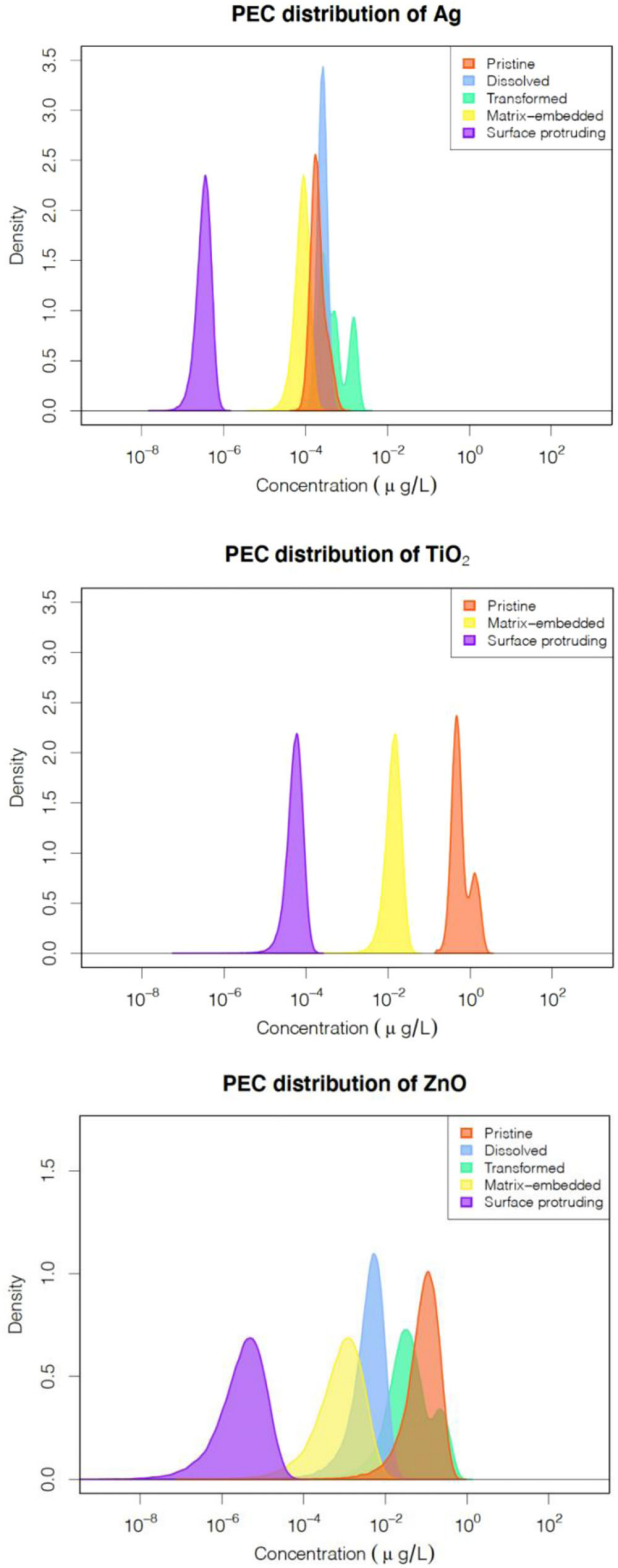
Histograms of the probability density distribution of the predicted environmental concentration values of different forms of nano‐Ag, nano‐TiO_2_, and nano‐ZnO. PEC = predicted environmental concentration.

Figure 1 shows the complete PEC probability distributions for the 3 ENMs in the freshwater environment. The PECs of the different forms of nano‐Ag in ascending order are surface‐protruding (3.5 × 10^–7^ µg/L) < matrix‐embedded (0.000088 µg/L) < pristine (0.00022 µg/L) < dissolved (0.00026 µg/L) < transformed (0.00064 µg/L). The largest fraction of nano‐Ag is the transformed form (56%), whereas the matrix‐embedded form was <0.1%. Nano‐TiO_2_ is present only in 2 forms: pristine and matrix‐embedded, with 99.99% of nano‐TiO_2_ existing as the pristine form. The PEC of surface‐protruding TiO_2_ (0.000058 µg/L) is 4 orders of magnitude lower than pristine nano‐TiO_2_ (0.70 µg/L). For nano‐ZnO, the PECs increase in the following order: surface‐protruding (5.1 × 10^–6^ µg/L) < matrix‐embedded (0.0013 µg/L) < dissolved (0.0045 µg/L) < transformed (0.070 µg/L) < pristine (0.098 µg/L) form. According to the mean of the PEC values, 56% of nano‐ZnO is present as pristine and 40% as transformed ZnO. The fractions of dissolved and surface protruding forms are 2.60 and 0.0029%, respectively.

### Hazard assessment

As shown in Table 1, a total of 1988 data points were obtained from the literature. The dissolved metals had the highest number of data points (dissolved Ag, 447; dissolved Zn, 1040), whereas the transformed ENMs had a limited number of data points (transformed Ag, 5; transformed ZnO, 3). In all cases except transformed Ag and transformed ZnO, there were statistically enough species to build PSSDs. For transformed Ag and transformed ZnO only the PNEC_*deter*_ could be calculated. The PNEC_*deter*_ of transformed Ag was only based on data from AgS. The data for transformed ZnO contained more diverse types of transformed forms (phosphate‐aged forms, i.e., Zn_3_[PO_4_]_2_ × 4H_2_O and Zn_3_[PO_4_]_2_) and a sulfidized form (i.e., ZnS), but the numbers of data points and species were limited. Chronic data were less available than acute data, with 33 and 30% being the highest percentages obtained for nano‐ZnO and pristine nano‐TiO_2_, respectively, whereas for dissolved Ag and Zn this fraction was only 3%. The complete data used in the hazard assessment are provided in Supplemental Data B.

**Table 1:** Overview of data used in the hazard assessment

	Nano‐Ag			Nano‐TiO_2_	Nano‐ZnO			
	Pristine	Dissolved	Transformed	Pristine	Pristine	Dissolved	Transformed	Total
No. of data	186	447	5	178	70	1040	3	1988
No. of species	29	44	3	28	19	169	1	245
No. of chronic data	22	9	1	53	23	36	0	192
	(12%)	(3%)	(20%)	(30%)	(33%)	(3%)	(0%)	

**Figure 2:**
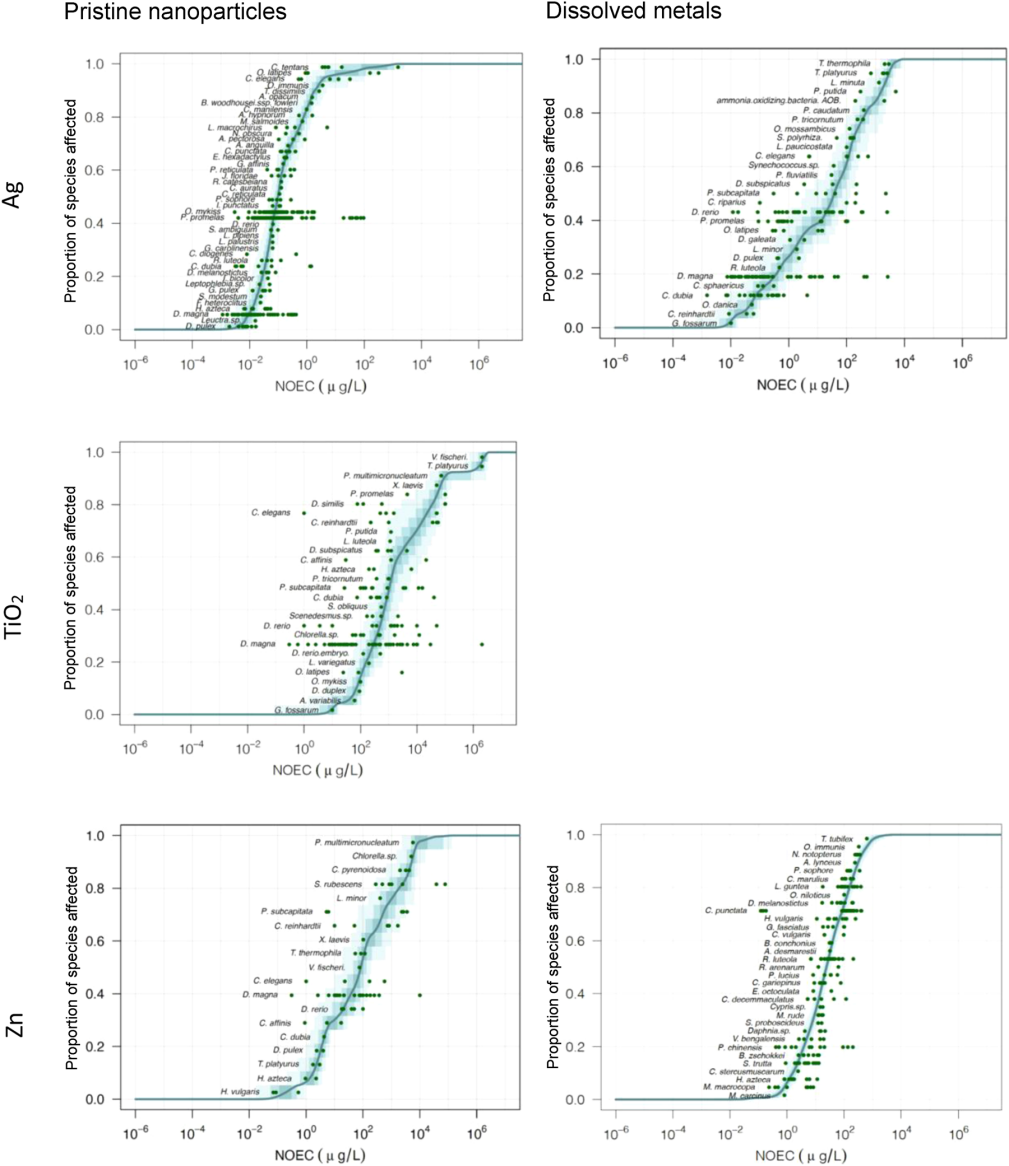
Probabilistic species sensitivity distributions (PSSDs) for pristine nano‐Ag and dissolved Ag (top), nano‐TiO_2_ (middle), and nano‐ZnO and dissolved Zn (bottom). Different shades display the percentiles of the theoretical no‐observed‐effect concentration (NOEC) distribution: lighter blue = 0 to 100%; medium blue = 5 to 95%; dark blue = 25 to 75%. The thick line represents the mean PSSD and the green dots, the NOECs after conversion with uncertainty factors.

The form‐specific PSSDs of the ENMs are shown in Figure 2. For pristine nano‐Ag, the most sensitive species with a mean of the NOEC values below the HC5 were *Gammarus fossarum* (arthropod) and *Chlamydomonas reinhardtii* (algae). The PSSD of dissolved Ag was built on the basis on 447 data points of 44 species. Three freshwater arthropods, *Daphnia pulex, Hyalella azteca*, and *Daphnia magna*, were found to be the most sensitive species. For nano‐TiO_2_, the PSSD was calculated only for the pristine form. The mean NOECs of *G. fossarum* (arthropod) and *Anabaena variabilis* (cyanobacterium) were lower than the HC5. The PSSD of pristine nano‐ZnO contains 70 data points of 19 species. *Hydra vulgaris*, a small freshwater hydroid, was found to be the only species whose mean NOEC was below the HC5. Dissolved Zn had the largest number of data points and species to build the PSSD (1040 data points of 169 species). The PSSDs were calculated with the complete data set; however, only one‐fifth of the species were randomly chosen and are displayed in Figure 2 to improve readability. Supplemental Data, Figure S1 provides a closer look at the lower limit (15%) of the PSSD, without omitting the name of the species and the chronic NOEC points. Eight species (*Etroplus maculatus, Cirrhinus mrigala, Barbus ticto, Oncorhynchus clarkii, Cottus bairdi, Mesocyclops hyalinus, Macrobrachium carcinus, and Branchiura sowerbyi*) were below the HC5 of dissolved Zn, including 3 trophic levels (5 species of fish, 2 species of crustacean, and 1 species of worm).

The PNEC values (equal to HC5) extracted from the PSSDs are summarized in Table 2. The deterministic PNEC values, specified by “deter” (e.g., transformed Ag_*deter*_ and transformed ZnO_*deter*_), are given in the column of the mean value. For the PNEC determination based on the PSSD approach, the 5th and 95th percentiles (Q_5_, Q_95_) are shown with the mean and the mode values.

**Table 2:** Summary of the predicted‐no‐effect concentration values for the different forms of nano‐Ag, nano‐TiO_2_, and nano‐ZnO (in micrograms per liter)

		Q_5_	Mean	Mode	Q_95_
Nano‐Ag					
Pristine		0.0084	0.028	0.036	0.059
Dissolved		0.0069	0.013	0.016	0.021
Transformed_*deter*_		—	6.8	—	—
Nano‐TiO_2_					
Pristine		9.7	41.8	56.8	78.1
Nano‐ZnO					
Pristine		0.057	0.22	0.22	0.47
Dissolved		0.65	0.85	0.84	1.1
Transformed_*deter*_		—	0.43	—	—

Q_5_ and Q_95_ = 5th and 95th percentiles, respectively.

The means of the PNECs of different forms of nano‐Ag in ascending order are dissolved (0.013 µg/L) < pristine (0.028 µg/L) < transformed_*deter*_ (6.8 µg/L). The PNECs of pristine nano‐Ag and dissolved Ag have the same order of magnitude. The mean PNEC of pristine nano‐TiO_2_ was determined to be 41.8 µg/L. Among the 3 different forms of nano‐ZnO, pristine nano‐ZnO was identified as the most toxic form to the environment (mean of PNEC, 0.22 µg/L). Transformed ZnO was determined to be the next most toxic form (PNEC_*deter*_ of transformed ZnO, 0.43 µg/L) with the same order of magnitude of effect concentration as pristine nano‐ZnO. The least toxic form was the dissolved form (mean of PNEC, 0.85 µg/L).

### Environmental risk assessment

For all forms of nano‐Ag, the PECs are lower than the PNECs of the respective forms. For pristine nano‐Ag and dissolved Ag, the PEC and PNEC differed by 2 orders of magnitude (Supplemental Data, Figure S2). For transformed Ag and surface‐protruding Ag, the differences between the PEC and PNEC are 3 orders of magnitude. In the case of pristine nano‐TiO_2_, the PNEC is 2 orders of magnitude higher than the PEC. However, the lower tail of the PNEC distribution overlaps with the upper tail of the PEC distribution (Supplemental Data, Figure S3). The fraction of the range of overlap compared to the whole range of these 2 distributions was determined to be 1.1%. For surface‐protruding TiO_2_, there is a difference of 6 orders of magnitude between the PEC and PNEC distributions. Among the 4 different forms of nano‐ZnO, the PEC and PNEC distributions of pristine nano‐ZnO overlap (Supplemental Data, Figure S4). The range of overlap is approximately 80%. The PEC and PNEC distributions of dissolved Zn and surface‐protruding ZnO do not overlap, and the mean of each distribution differs by more than 4 orders of magnitude. The PNEC_*deter*_ of transformed Zn corresponds to the 98.5th percentile of the PEC distribution of transformed Zn.

**Figure 3:**
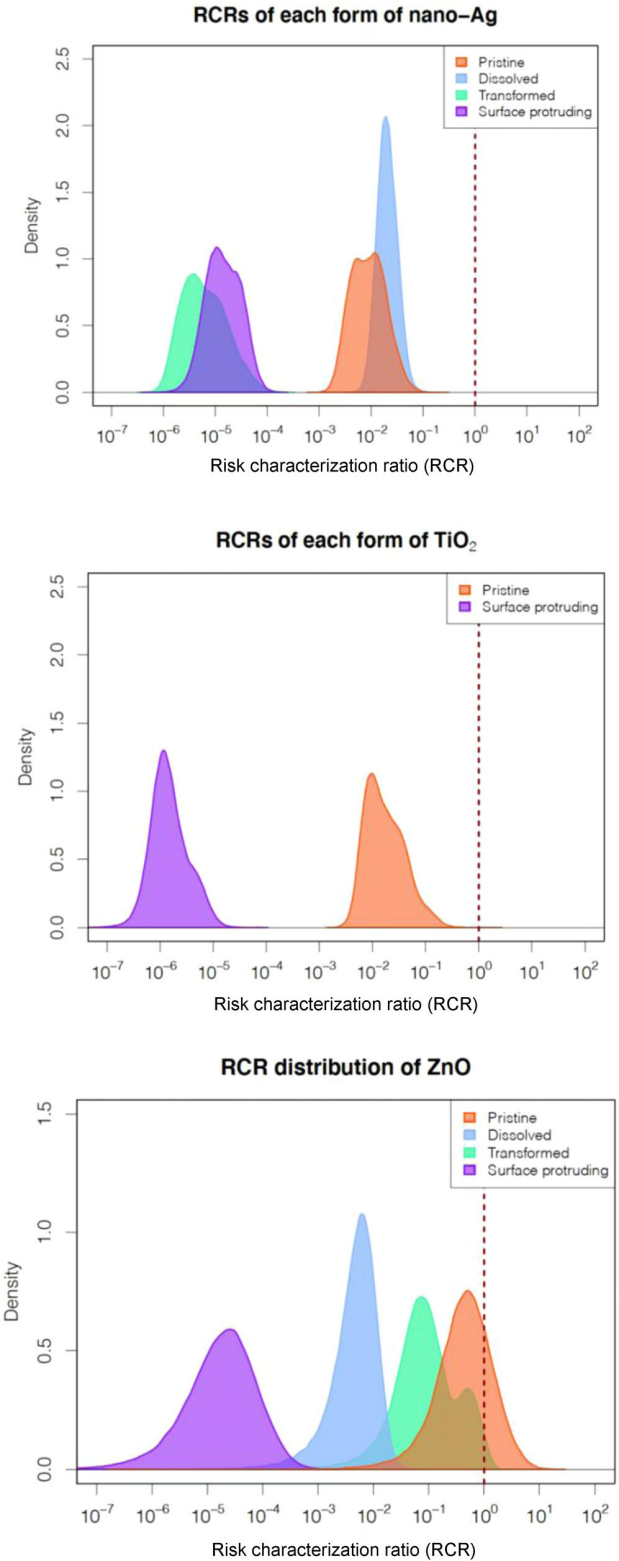
Histograms of the probability density distribution of the risk characterization ratio (RCR) of different forms of nano‐Ag, TiO_2_, and ZnO. The right side of the dashed line (red) represents the area of potential risks. The vertical line indicates the RCR of 1.

The distribution of the RCR was determined by dividing the PEC distribution by the PNEC distribution. The complete RCR distributions are shown in Figure 3; the mean, mode, Q_5_, and Q_95_ are compiled in Table 3. All RCRs of different forms of nano‐Ag are much lower than 1. The 5th and 95th percentiles of the RCR distributions span the ranges of 0.0027 to 0.032 for pristine, 0.010 to 0.043 for dissolved, 1.5 × 10^–6^ to 3.5 × 10^–5^ for transformed, and 4.1 × 10^–6^ to 4.9 × 10^–5^ for surface‐protruding forms (Table 3). Therefore, the RCR of different forms of nano‐Ag in ascending order were pristine < dissolved < surface‐protruding <transformed. The RCRs of the 2 forms of nano‐TiO_2_ are also lower than 1. The pristine form, which is the major form of nano‐TiO_2_, has a mean value <1 by 2 orders of magnitude. The mean RCR of surface‐protruding TiO_2_ was estimated to be 2.1 × 10^–6^ (Table 3).

**Table 3:** Risk characterization ratio of different forms of engineered nanomaterials

	Q_5_	Mean	Mode	Q_95_
Nano‐Ag				
Pristine	0.0027	0.012	0.012	0.032
Dissolved	0.010	0.023	0.018	0.043
Transformed	1.5 × 10^–6^	1.0 × 10^–5^	3.3 × 10^–6^	3.5 × 10^–5^
Surface‐protruding	4.1 × 10^–6^	1.9 × 10^–5^	9.5 × 10^–6^	4.9 × 10^–5^
Nano‐TiO_2_				
Pristine	0.0053	0.026	0.0097	0.081
Surface‐protruding	4.5 × 10^–7^	2.1 × 10^–6^	1.1 × 10^–6^	6.4 × 10^–6^
Nano‐ZnO				
Pristine	0	0.68	0.49	2.4
Dissolved	0	0.0054	0.0063	0.014
Transformed	0	0.16	0.075	0.70
Surface‐protruding	0	3.5 × 10^–5^	2.13 × 10^–5^	1.4 × 10^–4^

Q_5_ and Q_95_ = 5th and 95th percentiles, respectively.

For ZnO, 2 of the RCR distributions extend to values >1: 20% of the RCR values for pristine nano‐ZnO and 1.45% for transformed ZnO exceed 1. It should be noted that the RCR distribution of transformed ZnO was calculated by dividing the PEC distribution by a single PNEC_*deter*_ value because no PNEC_*PSSD*_ could be derived. The means of the RCRs of dissolved Zn and surface‐protruding ZnO are 1 order of magnitude and 5 orders of magnitude lower than 1, respectively (Table 3).

### Comparison of risk characterization methods

**Figure 4:**
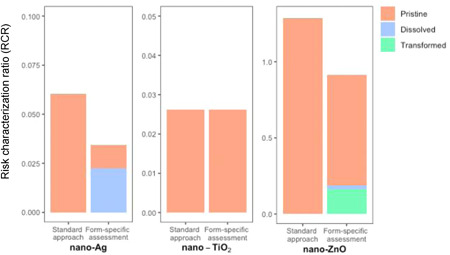
Comparison of the environmental risk assessment for nano‐Ag, nano‐TiO_2_, and nano‐ZnO using the standard generic approach for pristine engineered nanomaterials (left bar) and the summation of the form‐specific risk characterization ratio (right bar). The values of the surface protruding forms are not shown because they are too small to be visible. The calculation was done using the mean values presented in Table 3. The numerical values are given in Supplemental Data, Table S5.

The RCR was determined in a standard approach using the generic PEC value for the ENM and the PNEC of the pristine form using Equation 1. The results are presented in Figure 4 as “standard approach.” The overall form‐specific RCR was calculated by the PEC/PNEC summation approach and is shown in Figure 4 as “form‐specific assessment” using the mean values of the RCR. For nano‐Ag, RCR_standard_ was estimated to be roughly one‐third lower than RCR_form‐specific_. This decrease in risk is caused mainly by transformed Ag. According to the PNEC estimation, transformed Ag is the least hazardous form of nano‐Ag. However, more than half of nano‐Ag exists in the transformed form (56%) in freshwaters. As a consequence, the standard approach overestimates the risk of nano‐Ag by ignoring the form that demonstrates a low environmental hazard. There is almost no difference between RCR_standard_ and RCR_form‐specific_ for nano‐TiO_2_. This is mainly due to the high proportions of pristine nano‐TiO_2_ compared to matrix‐embedded TiO_2_ after release (99.99% exists in pristine form). Therefore, the consideration of matrix‐embedded TiO_2_ in the risk assessment did not alter the RCR value. The RCR of nano‐ZnO decreased when considering the released form (RCR_standard_, mean of 1.20; RCR_form‐specific_, mean of 0.85). Because an RCR of 1 is the regulatory trigger value, the different risk‐assessment methods characterize the environmental risk of nano‐ZnO differently (i.e., the standard approach identifies the environmental risk of nano‐ZnO to be not acceptable, whereas the form‐specific method concludes that it is slightly below 1).

## DISCUSSION

In the present study we combined the risk‐assessment approach for ENM based on the PEC/PNEC ratio with the recent advancements in quantifying environmental flows of different forms of ENM. The risk assessment based on the pristine form, which is the one normally targeted in hazard studies, was also the one on which previous risk assessments for ENMs were based (Wigger et al. 2020a). This means that for this form a large number of studies have been performed and that for the targeted ENM a good database exists to determine NOEC values. It is only for the dissolved metal ions that even more data are available because all metal risk assessments were based on this form. Only a few studies have quantified the effects of transformed ENM in a way that NOEC values could be determined. For Ag, only studies with AgS could be considered; for Zn, in addition phosphate‐aged forms were tested. Both AgS and ZnS have been identified as major forms after transformation of Ag and ZnO in wastewater (Kaegi et al. 2013; Ma et al. 2014; Brunetti et al. 2015).

In this context it is important to acknowledge that the PEC values we provide are based on the forms that are released to the environment and not on a fate modeling of ENM in the environment. The PEC values consider a worst case with no further sedimentation from water, a process that would decrease the concentration of ENM present in the water column. Because the present risk assessment only covers the aqueous freshwater environment, it can be considered a worst‐case assessment. Models such as NanoFate (Garner et al. 2017) and SimpleBox 4Nano (Meesters et al. 2014) can be used to derive more specific PEC values incorporating processes such as heteroagglomeration, sedimentation, and dissolution. Not included in these models are chemical transformation reactions (e.g., sulfidation). The results provided in the present study are therefore representative for a situation close to the emission source (e.g., the outlet of a wastewater‐treatment plant). Other processes that may affect the toxicity of the pristine ENM, such as formation of an eco‐corona, may also affect the hazard of the ENM and could subsequently also result in a changed environmental risk. The eco‐corona may both increase and decrease the toxicity of the ENM (Xu et al. 2020), although in most cases the eco‐corona reduced the interaction of ENM with the organisms. Such processes could be included in the risk assessment as soon as quantitative data for realistic environmental conditions are available and not just mechanistic studies that aim to investigate the underlying processes of eco‐corona effects under specific circumstances.

We also have to consider that the release assessment of the different forms is based on limited quantitative data and only presents an average behavior. Differences in the specific conditions during use and release could result in largely different distributions of the forms of release. Our assessment therefore constitutes a first step toward understanding the influence of transformation reactions on the environmental risks posed by ENM, but the results need to be treated with caution because in specific situations, both after release and later after additional environmental transformations, different form may exist compared to the generic assessment used in the present study.

One form of the ENM included in our assessment is the matrix‐embedded ENM. These materials could be handled in 2 ways: based on the currently available data on the toxicity of the ENM contained in matrix fragments, they could simply be omitted from the risk assessment. Amorim et al. (2018), for example, showed that for different ENM added to various matrices (e.g., epoxy, polyolefin, polyoxymethylene, and cement) the ENM were only partially exposed at the surface of the released fragments, suggesting that the matrix actually determined the toxicity of the fragments and not the ENMs. Because our assessment is targeted toward the ENM and does not include the matrix itself, it would be thus conceivable to just set the toxicity of matrix fragments to zero.

As an alternative, we decided to include an effect of surface‐protruding ENM by estimating their concentration (PEC value) and assuming that their toxic effects can be described by the pristine ENM toxicity. Schlagenhauf et al. (2015) quantified the amount of free CNTs and surface‐protruding CNTs when abrading a specific type of CNT–epoxy composite. This is so far the only study available that has quantified the amount of an ENM that is exposed on the surface of matrix fragments released by abrasion processes. However, the conversion factor describing the fraction of surface‐protruding ENM from their study is based on one specific matrix, a specific ENM (in their case, CNT) and a defined abrasion process (Taber abrader). The composition of the matrix, the type of ENM, and the size of the released matrix particle will all influence the fraction of particles exposed on the surface. However, this fraction will also be small, and thus, the PEC of surface‐protruding ENM will be much lower than for pristine ENM. In the overall risk assessment shown in Figure 4 the contribution by the surface‐protruding ENM is not visible for any of the 3 ENMs considered. Even increasing the PEC of matrix‐embedded ENM by a factor of 10 would not change the overall risk to any significant degree. This PEC would need to increase 92, 612, and 1185 times for Ag, TiO_2_, and Zn, respectively, to result in a 5% change of the RCR.

For 2 metals, Ag and Zn, the present study provides PNEC values for both the pristine form (nano‐Ag and nano‐ZnO) as well as the dissolved metal ion. For Ag, the dissolved Ag PNEC is a factor of 2 lower than the pristine nano‐Ag PNEC, in line with the expectation that pristine nano‐Ag is less toxic than dissolved Ag (Notter et al. 2014; Skjolding et al. 2016; Salieri et al. 2020). For Zn, however, the PNEC for pristine nano‐ZnO is 4 times lower than for dissolved Zn, which is significant (*p* < 2.2e‐16 by t‐test). Notter et al. (2014) had observed in their meta‐analysis of data on dissolved and nano‐toxicity for the same metal that Zn had the highest proportion of data points where under identical exposure conditions the NP had a higher toxicity than the dissolved metal ion. One explanation for the lower PNEC may also be that for dissolved Zn only 3% of the NOEC values were chronic data and thus an uncertainty factor (UF_t_) was used to transform acute into chronic NOECs. The PSSDs between chronic and acute toxicity were compared for ENMs by Wigger et al. (2020b), who showed that the modal value of 10 for UF_t_ is reasonable. However, this may be different for dissolved metals, and thus, an additional uncertainty is introduced into the assessment by conversion to a chronic NOEC. With 169 species the dissolved Zn PSSD is very extensive and based on a very large set of studies, thus giving confidence in the evaluation. Although based on fewer data, the pristine nano‐ZnO PSSD is also very complete with 19 species.

The present study derived PNECs based on the most recent data set, but we can still compare the obtained values with those of previous studies (see Supplemental Data, Table S6). Wigger et al. (2020b) and Wigger and Nowack (2019) used the same PSSD+ method to derive PNEC values for nano‐TiO_2_ and nano‐Ag. The value for nano‐Ag was 0.08 µg/L in Wigger et al. (2020b) compared to 0.028 µg/L in the present study. The present study added newer data that reported higher toxicity, such as for *Ochromonas danica* and *Chlamydomonas reinhardtii*. A PNEC value of 0.017 µg/L was obtained by Coll et al. (2016) using an earlier version of the PSSD method and based on a much more restricted data set that was available 4 yr ago. Chen et al. (2018) used a different approach to obtain the PNEC value. These authors computed species sensitivity distributions (SSDs) based on NOEC values by generating a maximum‐likelihood fitting of univariate distributions with the 95% confidence interval of the regressions by the strategy of parametric bootstrap. They obtained a value of 0.36 µg/L, but their method did not consider a specific conversion factor for the exposure time (UF_t_); thus, their analysis was based on NOEC values that are a mix of acute and chronic data. In our data set only 20% of the data are originally chronic NOEC values, and thus, 80% have been transformed using UF_t_, resulting in much lower values as input for the SSD. There is a similar issue with the SSD for nano‐Ag performed by Garner et al. (2015), who based the evaluation of EC50 values, which are again 10 times higher than NOEC values and, as a consequence, their reported HC5 is much higher.

For nano‐TiO_2_ the PNEC value from Coll et al. (2016) was 15.7 µg/L, and the ones from Wigger and Nowack (2019) were 38 µg/L for nano‐anatase and 33 µg/L for nano‐rutile. The value of 41 µg/L from the present study is comparable, and the inclusion of additional newer data therefore did not change the PNEC value to any significant degree.

The mean PNEC of nano‐ZnO from the present study was 5 times lower than the one obtained by Coll et al. (2016). The present study contains new data on more sensitive species, such as *Hydra vulgaris* and *Hyalella azteca*. The value published by Chen et al. (2018) is another factor of 5 higher, but the same issue also applies to this material. Overall, we can therefore state that PNEC values obtained by different studies tend to gravitate toward the same value when differences in the procedure used to obtain the NOEC values are considered.

To calculate the overall risk by the different forms, a cumulative risk assessment needs to be performed (Moretto et al. 2017). There are several approaches suggested in the ERA literature for mixtures, such as the PEC/PNEC summation and the toxic unit summation for trophic levels (European Chemicals Agency 2017). The present study is based on the PEC/PNEC summation approach, which is the least realistic but the most conservative among the 3 approaches. The PEC/PNEC summation approach is the quantification of the independent random events of responses to individual components (e.g., pristine ENM). This approach is considered to be a tier 1 assessment (European Chemicals Agency 2017) and is therefore a reasonable first step.

The value of RCR_form‐specific_ is well below 1 for nano‐Ag and nano‐TiO_2_, indicating only a limited environmental risk in freshwaters for these 2 ENMs. For nano‐Ag the cumulative assessment using the form‐specific RCR resulted in lower values than the standard approach just using the pristine ENM. This is reasonable because the transformed form of nano‐Ag is known to be of much lower toxicity than the pristine form (Levard et al. 2012; He et al. 2019). The dissolved form of Ag also contributes to the overall risks, and because the PNEC of dissolved Ag is lower than that of nano‐Ag, including this form can increase again the overall RCR. In soils, the effect of transformation on the RCR would be even greater because only 3% of Ag is expected to be released in pristine form into soils (Adam et al. 2018). The matrix‐embedded form is 15% and the transformed form 82% of the released nano‐Ag (Adam et al. 2018). For soil risk assessment, using a form‐specific approach is therefore even more important than for freshwaters because the transformed form with its much lower toxicity has a higher share of the released nano‐Ag.

For nano‐TiO_2_ there is no difference between the standard approach and the cumulative approach. Transformation into other mineral forms does not play a role for nano‐TiO_2_ released into freshwater (Adam et al. 2018), and the matrix‐embedded form has a very low toxicity. For soils the matrix‐embedded form was estimated to be 9% of the total release (Adam et al. 2018), so 3 times larger than for freshwaters. This would still result in only a slight decrease in environmental risks for soils.

For nano‐ZnO, the form‐specific assessment resulted in a slightly reduced environmental risk. The RCR decreased from slightly above 1 to slightly below 1. This NM has previously been identified as the one where the RCR is closest to 1 and was identified as a candidate ENM which should be further investigated (Coll et al. 2016). Given the high reactivity of ZnO and the limited stability under environmental conditions, it is expected that rapid transformation into other mineral forms such as Zn‐carbonate or Zn‐phosphate occurs. These Zn forms formed under natural conditions have a lower toxicity than pristine ZnO (Wang et al. 2017).

## CONCLUSIONS

Recently, the first models providing quantitative data on flows of different form of ENM to the environment have become available, and the present study reports how this information can be used for a form‐specific ERA of nano‐TiO_2_, nano‐Ag, and nano‐ZnO. For some forms of the ENM (e.g., pristine and dissolved forms) there are sufficient ecotoxicological data available to perform a hazard assessment using PSSDs. For transformed nano‐Ag and nano‐ZnO only a few studies are available, and thus, only a deterministic PNEC could be obtained. The matrix‐embedded form was included in our assessment by considering the concentration of surface‐exposed ENM and assuming that their toxicity can be described by the pristine ENM. Using this approach, we could show that the RCR for nano‐Ag and nano‐ZnO considering the released forms was smaller than for the standard approach that based the whole risk assessment only on the pristine form. For nano‐TiO_2_ the 2 approaches resulted only in very small differences because most of the nano‐TiO_2_ released into surface waters is predicted to be present in pristine form. These results show that for each ENM the form‐specific risk assessment needs to be performed separately because it is determined by the relative distribution of the released forms and the differences between the effects of different forms. However, overall, we can expect that using a form‐specific approach will decrease the RCR because transformed and matrix‐embedded forms usually have lower toxicity than the pristine ENM.

## Supplemental Data

The Supplemental Data include parameters used to calculate PEC values and UFs used for the hazard assessment. They also include a table summarizing PEC values, histograms of the distributions of PEC and PNEC values, and a summary of RCRs. The whole data set used to determine the form‐specific PNEC is shown in a separate Excel sheet. The Supplemental Data are available on the Wiley Online Library at https://doi.org/10.1002/etc.5146.

## Supporting information

This article includes online‐only Supplemental Data.

Supporting information.

Supporting information.

## Data Availability

Data, associated metadata, and calculation tools are available from the corresponding author (nowack@empa.ch).
