# Supplementary material for: Form‐Specific and Probabilistic Environmental Risk Assessment of 3 Engineered Nanomaterials (Nano‐Ag, Nano‐TiO2, and Nano‐ZnO) in European Freshwaters
Source: Environ Toxicol Chem. 2021 Aug 4;40(9):2629–39. doi: 10.1002/etc.5146 (PMC8457094; doi:10.1002/etc.5146)
Supplement: Supplementary file 1 — Supporting information. [file ETC-40-2629-s002.pdf]

1. PEC value calculation

This study considered the residence time of water for the European situation as a simplified method to estimate cumulative quantity in the freshwater compartment. The residence time of water of 40 days is recommended by (ECHA, 2010) assuming a steady state between the annual addition and removal of the freshwater. Therefore, the annual mass estimation of different forms of ENMs were multiplied by a conversion factor of 40/365.

Table S1. Summary of parameters to derive the volume of freshwater in Europe

	value	unit	Reference
Surface area of EU	4479384	km^2	The geography of the European Union, 2019, https://en.wikipedia.org/wiki/Geography_of_the_European_Union
Share of water area in EU	3	%	(ECHA, 2010)
Average depth of freshwater in EU	3	m	(ECHA, 2010)
Transformation factor from km^2 to m^2	10^6	-	-
Transformation factor from m^3 to litre	1000	-	-
Volume of freshwater in Europe in litre	$4479384 \times 0.03 \times 10^6 \times 3 \times 1000$		

2. Hazard assessment: Uncertainty factors

In most cases, the article from which the toxicity endpoint was originated had clarified whether the study was for an acute or chronic toxicity. There were a few cases where authors did not clarify whether the experiment was for acute or chronic toxicity. In such cases, other publications on the same species were reviewed to determine the acute and chronic toxicity of the experiment. The cut-off value for amphibian toxicity studies was set to be 14 days based on previous research (Chai, Wang, Deng, & Zhao, 2014; Farquharson, Wepener, & Smit, 2016; Fridman, Corró, & Herkovits, 2004; Maher, Werner, & Denver, 2013). For bacteria, the cut-off of 24 hours was selected as a reasonable criteria given the short life cycle of organisms. (Blaschke, Paschke, Rensch, & Schüürmann, 2010). The lists of updated UFs are shown in Tables S2 and S3. Although the cut-off value of *Ceriodaphnia dubia* was provided in the original list of (Wigger and Nowack, 2019), this study applied the same cut-off criteria to all *Ceriodaphnia* species according to (Versteeg, Stalmans, Dyer, & Janssen, 1997).

Table S2. Uncertainty factors used to convert acute to chronic values (UF_t)[a]

Type of organism	Exposure time (days)	UF_t modal value
Algae	< 3	10
	≥ 3	1
Plants[b]	< 28	10
	≥ 28	1
*Crustacea[c]	< 21	10
	≥ 21	1
Mollusca	< 28	10
	≥ 28	1
Fish	< 28	10
	≥ 28	1
*Amphibian	< 14	10
	≥ 14	1
*Bacteria	< 1	10
	≥ 1	1

*indicates an update compared to the original publication of Wigger et al. (2019) – see above text for references.

[a] UFt = uncertainty factor for transformation of acute toxicity into chronic toxicity.

[b] For *Lemna sp.*, the cut-off value of 2.5 days was set to distinguish acute from chronic effect, according to OECD Test Guideline 221.

[c] According to the testing guideline of US EPA on *C. dubia* and Versteeg et al. (1997), the cut-off value of 7 days were considered for all *Ceriodaphnia* species.

Table S3. Uncertainty factor used to convert a specific dose descriptor into a NOEC[a]

Type of dose descriptor	UF_{dd} modal value
LC_{25-50}; EC_{25-50}; IC_{25-50}	10
LC_{20}; EC_{20}; IC_{20}	2
LOEC	2
MIC	2
LC_{10}; EC_{10}; IC_{10}	1
HONEC	1

[a] LC = lethal concentration; EC = effect concentration; IC = inhibitory concentration; LOEC = lowest observed effect concentration; MIC = minimum inhibitory concentration; HONEC = highest observed no-effect concentration; UF_{dd} = uncertainty factor for the conversion of dose descriptor

3. Results

Table S4. Predicted environmental concentrations for the different forms of nano-Ag, nano-TiO_2 and nano-ZnO (in µg/L).

	Q_5	Mean	Mode	Q_{95}
nano-Ag				
Pristine	0.00011	0.00022	0.00017	0.00045
Dissolved	0.00016	0.00026	0.00027	0.00039
Transformed	0.00017	0.00064	0.00027	0.0018
Matrix-embedded	$3.8 \cdot 10^{-5}$	$8.8 \cdot 10^{-5}$	$9.0 \cdot 10^{-5}$	0.00015
Surface protruding	$1.5 \cdot 10^{-7}$	$3.5 \cdot 10^{-7}$	$3.6 \cdot 10^{-7}$	$6.0 \cdot 10^{-7}$
nano-TiO_2				
Pristine	0.30	0.70	0.47	1.7
Matrix-embedded	0.0057	0.014	0.015	0.026
Surface protruding	$2.3 \cdot 10^{-5}$	$5.8 \cdot 10^{-5}$	$5.9 \cdot 10^{-5}$	$1.0 \cdot 10^{-4}$
nano-ZnO				
Pristine	0	0.098	0.11	0.27
Dissolved	0	0.0045	0.0051	0.012
Transformed	0	0.070	0.032	0.30
Matrix-embedded	0	0.0013	0.0013	0.0044
Surface protruding	0	$5.1 \cdot 10^{-6}$	$5.0 \cdot 10^{-6}$	$1.8 \cdot 10^{-5}$

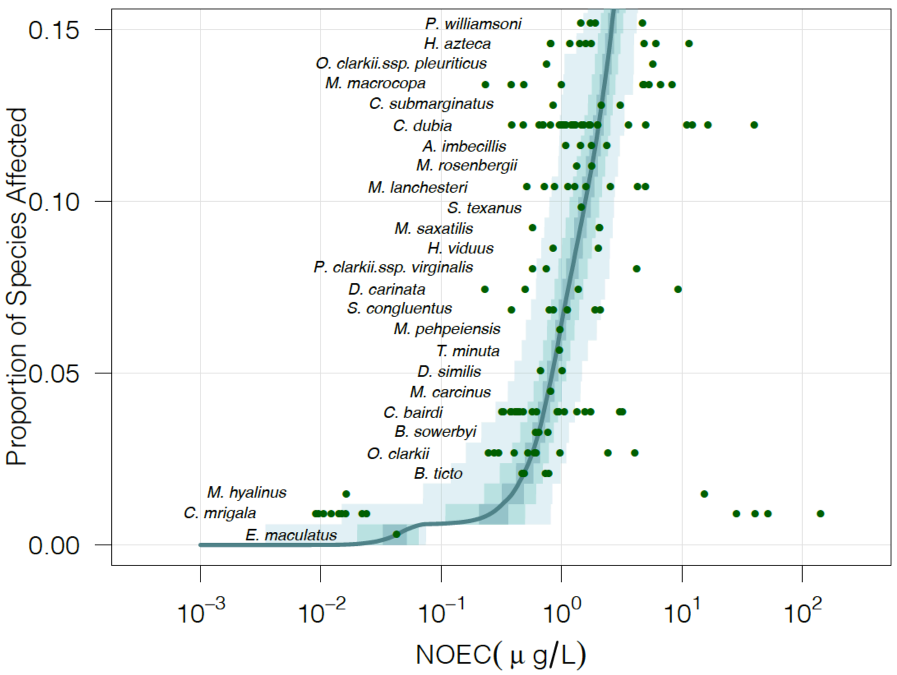

Figure S1. Lower part (15%) of the probabilistic species sensitivity distribution of dissolved Zn. The different shades display the percentiles of the theoretical NOEC distribution: the 0-100% (lighter blue), 5-95% (medium blue) and 25-75% percentiles (dark blue). The thick line represents the mean PSSD and the green dots the NOECs after conversion with UFs.

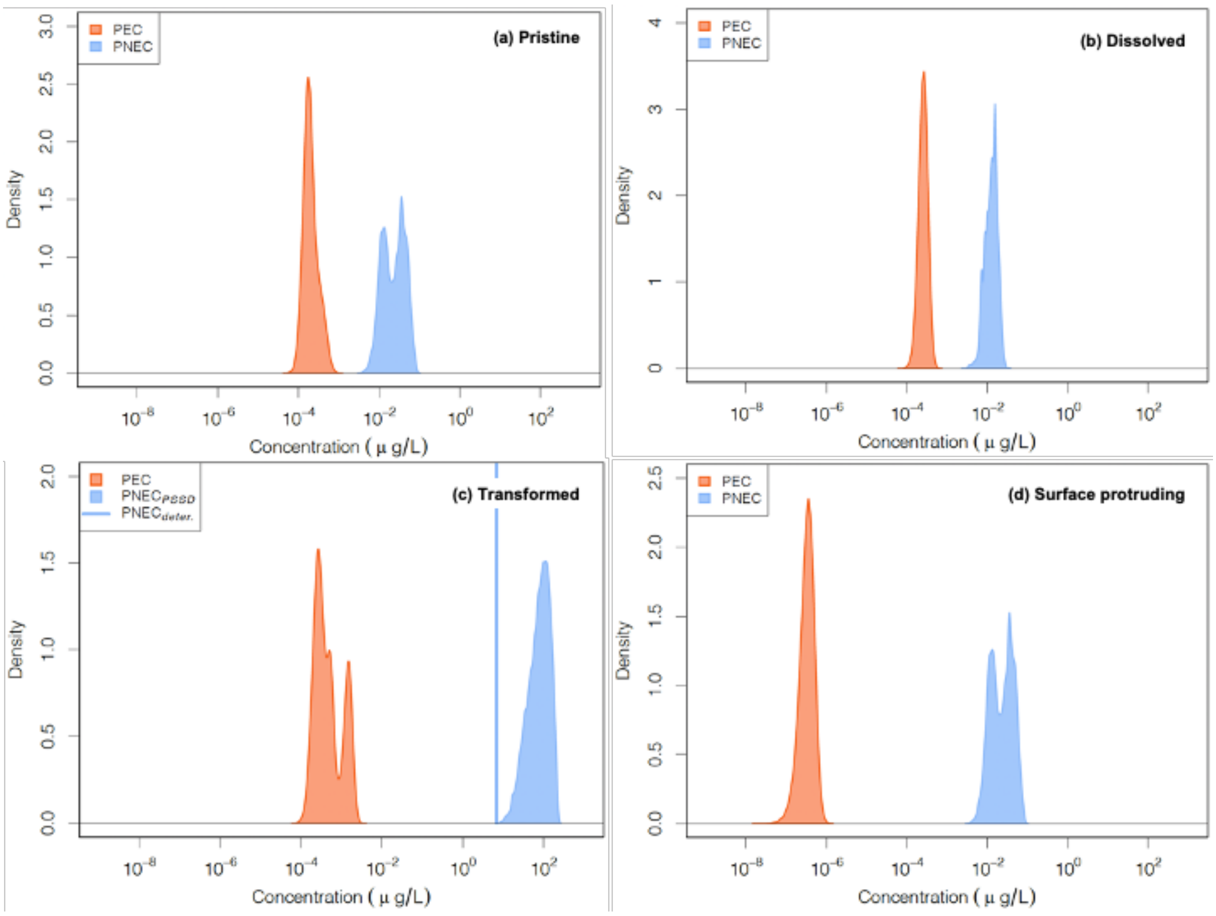

Figure S2. Histogram of the distributions of PEC and PNEC of different forms of nano-Ag.

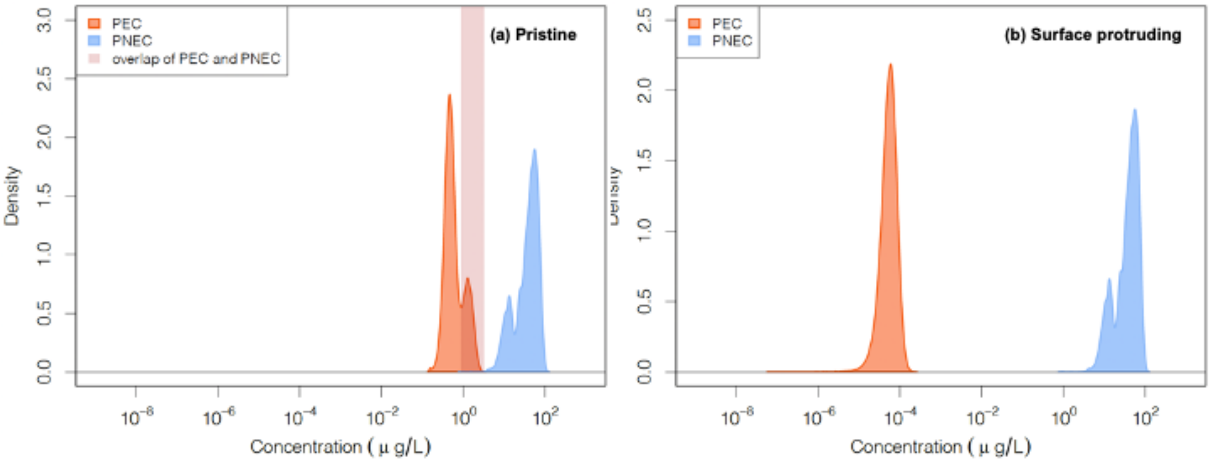

Figure S3. Histogram of the distributions of PECs and PNECs of different forms of nano-TiO_2. The red rectangle shows the range where an overlap of PEC and PNEC exists.

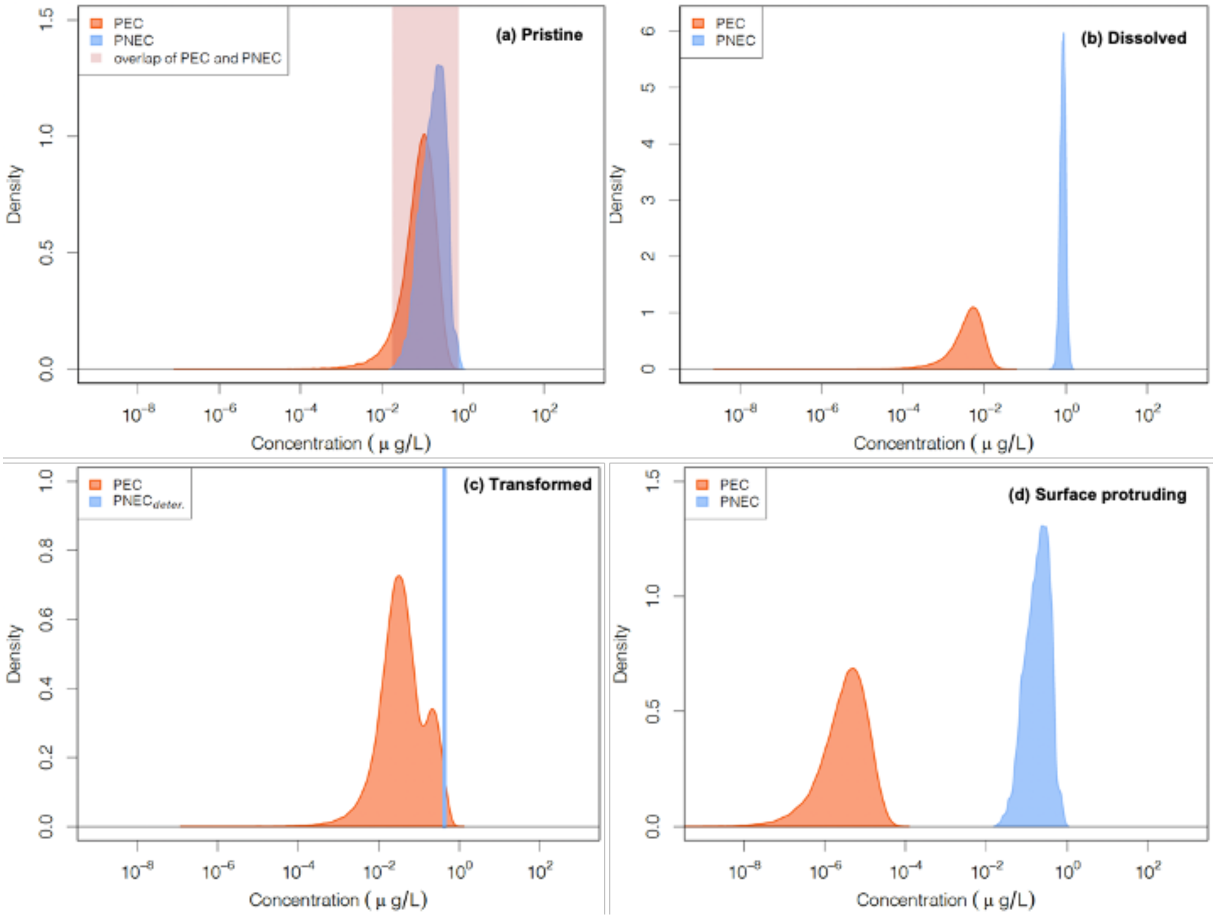

Figure S4. Histogram of the distributions of PECs and PNECs of different forms of nano-ZnO. The red rectangle shows the range where an overlap of PEC and PNEC exists.

Table S5. Summary of $RCR_{standard}$ and $RCR_{form\text{-}specific}$

		Q_5	Mean	Mode	Q_{95}
Nano-Ag	$RCR_{standard}$	0.013	0.061	0.055	0.17
	$RCR_{form\text{-}specific}$	0.016	0.034	0.031	0.063
Nano-TiO_2	$RCR_{standard}$	0.0053	0.026	0.0097	0.082
	$RCR_{form\text{-}specific}$	0.0053	0.026	0.0097	0.082
Nano-ZnO	$RCR_{standard}$	0.087	1.2	0.74	4.1
	$RCR_{form\text{-}specific}$	0.048	0.86	0.71	2.7

Table S6. Comparison of PNEC values from this study and from the literature

	Method	Mean
Nano-Ag		
Current study	PSSD+ (pristine)	0.028
(Coll et al., 2016)	PSSD	0.017
(Chen et al., 2018)	SSD	0.36
(Wigger and Nowack, 2019)	PSSD+	0.08
Nano-TiO_2		
Current study	PSSD+ (pristine)	41.4
(Coll et al., 2016)	PSSD	15.7
(Chen et al., 2018)	SSD	190.0
(Wigger and Nowack, 2019)	PSSD+ (anatase)	38.0
(Wigger and Nowack, 2019)	PSSD+ (rutile)	33.0
Nano-ZnO		
Current study	PSSD+ (pristine)	0.22
(Coll et al., 2016)	PSSD	1.0
(Chen et al., 2018)	SSD	5.1

References

Blaschke, U., Paschke, A., Rensch, I., & Schüürmann, G. (2010). Acute and chronic toxicity toward the bacteria Vibrio fischeri of organic narcotics and epoxides: Structural alerts for epoxide excess toxicity. *Chemical Research in Toxicology*, *23*(12), 1936–1946.

Chai, L., Wang, H., Deng, H., & Zhao, H. (2014). Chronic exposure effects of copper on growth , metamorphosis and thyroid gland , liver health in Chinese toad, Bufo gargarizans tadpoles. *Chemistry and Ecology*, *30*(7), 589–601.

Chen, G., Peijnenburg, W.J.G.M., Xiao, Y., Vijver, M.G., 2018. Developing species sensitivity distributions for metallic nanomaterials considering the characteristics of nanomaterials, experimental conditions, and different types of endpoints. Food and Chemical Toxicology 112, 563-570.

Coll, C., Notter, D., Gottschalk, F., Sun, T.Y., Som, C., Nowack, B., 2016. Probabilistic environmental risk assessment of five nanomaterials (nano-TiO2, nano-Ag, nano-ZnO, CNT, Fullerenes). Nanotoxicology 10, 436-444.

ECHA, 2010. Guidance on information requirements and chemical safety assessment Chapter R.16: Environmental Exposure Estimation, European Chemicals Agency.

Farquharson, C., Wepener, V., & Smit, N. J. (2016). Acute and chronic effects of acidic ph on four subtropical frog species. *Water SA*, *42*(1), 52–62.

Fridman, O., Corró, L., & Herkovits, J. (2004). Estradiol uptake, toxicity, metabolism, and adverse effects on cadmium-treated amphibian embryos. *Environmental Health Perspectives*, *112*(8), 862–866.

Maher, J. M., Werner, E. E., & Denver, R. J. (2013). Stress hormones mediate predator-induced phenotypic plasticity in amphibian tadpoles. *Proceedings of the Royal Society B: Biological Sciences*, *280*(1758), 20123075.

Versteeg, D. J., Stalmans, M., Dyer, S. D., & Janssen, C. (1997). Ceriodaphnia and daphnia: A comparison of their sensitivity to xenobiotics and utility as a test species. *Chemosphere*, *34*(4), 869–892. https://doi.org/10.1016/S0045-6535(97)00014-3

Wigger, H., Nowack, B., 2019. Material-specific properties applied to an environmental risk assessment of engineered nanomaterials: implications on grouping and read-across concepts. Nanotoxicology, 1-21.
